# The complete chloroplast genome sequence of *Artocarpus hypargyreus*

**DOI:** 10.1080/23802359.2020.1715293

**Published:** 2020-01-27

**Authors:** Dong-Lin Li, Juma Gul, Jin-Ming He

**Affiliations:** a College of Ying-Tong Agricultural Science and Engineering, Shaoguan University, Shaoguan, China;; b Ministry of Education Key Laboratory for Ecology of Tropical Islands, College of Life Sciences, Hainan Normal University, Haikou, China

**Keywords:** *Artocarpus hypargyreus*, Moraceae, chloroplast genome, phylogenomic tree

## Abstract

The complete chloroplast genome sequences of *Artocarpus hypargyreus* was reported in this study. The length of the sequence was 160,952 bp in length with a large single copy (LSC) region of 89,476 bp, the small single copy (SSC) region of 20,070 bp and two inverted repeat (IR) regions of 25,703 bp. The complete genome contains 129 genes including 84 protein-coding genes, 8 rRNA genes and 37 tRNA genes. Phylogenetic analysis of the Moraceae based on 8 plastome sequence shows that *A. hypargyreus* is most related to *Morus cathayana*.

*Artocarpus hypargyreus* Hance is an endangered species, which belongs to the genus *Artocarpus* in the family Moraceae (Fu 1992). It is endemic to China, and only scattered in evergreen broad-leaved forests in Guangdong, Fujian, Jiangxi, Hunan, and Yunnan (Li et al. 2011). *A. hypargyreus* has high economic value and is widely used in our life. Its wood can be used as furniture; its fruits and seeds can be eaten raw, or be used as raw materials for candied fruit or beverages (Shen 2011). The roots of *A. hypargyreus* are widely used in northern Guangdong and western Yunnan for the treatment of rheumatoid arthritis and chronic back pain (Ouyang et al. 2010). In recent years, research on *A. hypargyreus* has focused on the chemical composition (He et al. 2016), screening and mechanism of effective medicinal parts (Shen et al. 2011), community structure (Tan et al. 2017), breeding technology (Li et al. 2010) and genetic diversity (Fan 2010). In this study, we reported and characterized the complete chloroplast genome sequence of *A. hypargyreus* to contribute to further phylogenetical and protective studies of this plant.

The fresh leaves of *Artocarpus hypargyreus* was collected from Xiafu village (:25°0′33″N, 113°42′06″E, Altitude: 106 m) in Shaoguan, Guangdong, China. And the Voucher specimens were deposited in the Herbarium of Shaoguan university, the accession number is Li-201904. Total DNA was extracted from the fresh young leaves using the Plants Genomic DNA Kit (DP305, Tiangen Biotech Co., Ltd., Beijing, China). The plastome sequences was generated using Illumina HiSeq 2500 platform (Illumina Lnc., San Diego, CA, USA). In total, 5.5 Gb raw reads were obtained. The filtered reads were assembled with the program NOVOPlasty 3.1 (Dierckxsens et al. 2017) with a part of *rbcL* gene of *Antiaris toxicaria* (NC 042884), and then the sequence of *A. hypargyreus* was annotated using DOGMA (Wyman et al. 2004). The annotated sequence was submitted to NCBI, the accession number is MN720648.

The complete chloroplast genome of *A. hypargyreus* is 160,952 bp in length, including a large single-copy (LSC) region of 89,476 bp, the small single copy region of 20,070 bp, and two inverted repeat (IR) regions of 25703 bp. The complete genome of *A. hypargyreus* contains 129 genes, including 84 protein-coding genes, 8 rRNA genes and 37 tRNA genes, the GC content of this genome was 35.80%.To further investigate the phylogenetic position of *A. hypargyreus* in Moraceae family, 8 of complete chloroplast genomes in family was download from NCBI, and then the maximum-likelihood (ML) phylogenetic tree was generated by MEGA 7.0 (Kumar et al. 2016), with *Antiaris toxicaria* as outgroup. The results in Figure 1 shows that *A. hypargyreus* is closed to *Morus cathayana*. Our study here could be further applied for the evolutionary and phylogenetic studies of this endangered plant.

**Figure 1.**
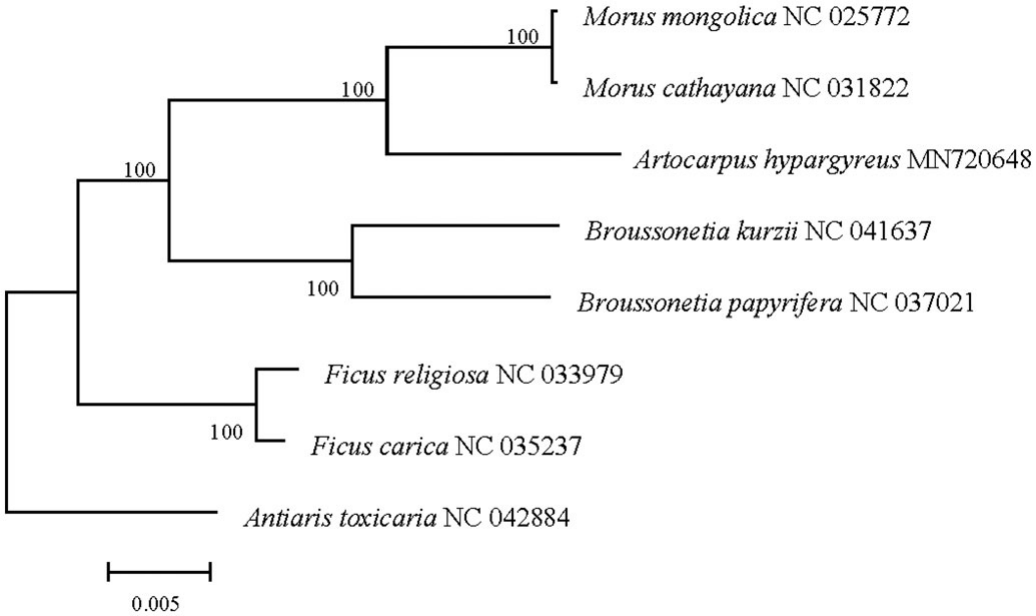
Maximum likelihood tree based on the sequences of eight complete chloroplast genomes. Numbers in the nodes were bootstrap values from 1000 replicates. Scale in substitutions per site.

## References

[CIT0001] Dierckxsens N, Mardulyn P, Smits G. 2017. NOVOPlasty: de novo assembly of organelle genomes from whole genome data. Nucleic Acids Res. 45(4):e18. 28204566 10.1093/nar/gkw955 PMC5389512

[CIT0002] Fan FR. 2010. Genetic diversity of endangered *Artocarpus hypargyreus*. J Zhejiang Forestry Coll. 27(2):266–271.

[CIT0003] Fu LG. 1992. China plant red book—rare and endangered plants. Beijing: Science Pres.

[CIT0004] He SY, Lu HX, Li M, Liu N, Zhou LY. 2016. Study on Chemical Constituents of Stems from *Artocarpus hypargyreus* Hance. J Yulin Teachers Coll. 37(5):99–101.

[CIT0005] Kumar S, Stecher G, Tamura K. 2016. MEGA7: molecular evolutionary genetics analysis Version 7.0 for bigger datasets. Mol Biol Evol. 33(7):1870–1874. 27004904 10.1093/molbev/msw054 PMC8210823

[CIT0006] Li GY, Xu PL, Chen GQ. 2011. Research on the tissue culture of endangered plant species *Artocarpus hypargyreus* hance. Trop Forestry. 39(3):24–29.

[CIT0007] Li GY, Xu PL, Chen GQ. 2010. A study of cultivation technique on seedling breeding of endangered plant *Artocarpus hypargyreus*. Trop Forestry. 38(3):23–24 + 16.

[CIT0008] Ouyang S, Shen ZJ, Pan LN. 2010. Active fraction selection of *Artocarpus hypargyraea* and its mechanism of anti-inflammatory and Analgesic Effects. Chin Tradit Herbal Drugs. 41(11):1850–1853.

[CIT0009] Shen QT. 2011. Germination physiology of endangered species *Artocarpus hypargyreus*. J Northwest Forestry Univ. 26(2):111–113.

[CIT0010] Shen ZJ, Qiao YD, Ouyang S, Pan LN. 2011. Active fraction selection of *Artocarpus hypargyraea* and its mechanism of anti-rheumatoid arthritis. Chin Tradit Herbal Drugs. 42(9):1792–1795.

[CIT0011] Tan WZ, Xu HL, Chen YM, Zhao WY, Zan QJ, Liao WB. 2017. Structure and succession of *Artocarpus hypargyreus* community in Neilingding Island, Guangdong. J South China Agric Univ. 38(2):99–105.

[CIT0012] Wyman SK, Jansen RK, Boore JL. 2004. Automatic annotation of organellar genomes with DOGMA. Bioinformatics. 20(17):3252–3255. 15180927 10.1093/bioinformatics/bth352

